# Oxidative Phosphorylation Is Dysregulated Within the Basocortical Circuit in a 6-month old Mouse Model of Down Syndrome and Alzheimer’s Disease

**DOI:** 10.3389/fnagi.2021.707950

**Published:** 2021-08-19

**Authors:** Melissa J. Alldred, Sang Han Lee, Grace E. Stutzmann, Stephen D. Ginsberg

**Affiliations:** ^1^Center for Dementia Research, Nathan Kline Institute, Orangeburg, NY, United States; ^2^Departments of Psychiatry, New York University Grossman School of Medicine, New York, NY, United States; ^3^Center for Biomedical Imaging and Neuromodulation, Nathan Kline Institute, Orangeburg, NY, United States; ^4^Department of Child and Adolescent Psychiatry, New York University Grossman School of Medicine, New York, NY, United States; ^5^Center for Neurodegenerative Disease and Therapeutics, Discipline of Neuroscience, Rosalind Franklin University/The Chicago Medical School, North Chicago, IL, United States; ^6^Neuroscience & Physiology, New York University Grossman School of Medicine, New York, NY, United States; ^7^NYU Neuroscience Institute, New York University Grossman School of Medicine, New York, NY, United States

**Keywords:** oxidative phosphorylation, basal forebrain, Down syndrome, Alzheimer’s disease, selective vulnerability

## Abstract

Down syndrome (DS) is the primary genetic cause of intellectual disability (ID), which is due to the triplication of human chromosome 21 (HSA21). In addition to ID, HSA21 trisomy results in a number of neurological and physiological pathologies in individuals with DS, including progressive cognitive dysfunction and learning and memory deficits which worsen with age. Further exacerbating neurological dysfunction associated with DS is the concomitant basal forebrain cholinergic neuron (BFCN) degeneration and onset of Alzheimer’s disease (AD) pathology in early mid-life. Recent single population RNA sequencing (RNA-seq) analysis in the Ts65Dn mouse model of DS, specifically the medial septal cholinergic neurons of the basal forebrain (BF), revealed the mitochondrial oxidative phosphorylation pathway was significantly impacted, with a large subset of genes within this pathway being downregulated. We further queried oxidative phosphorylation pathway dysregulation in Ts65Dn mice by examining genes and encoded proteins within brain regions comprising the basocortical system at the start of BFCN degeneration (6 months of age). In select Ts65Dn mice we demonstrate significant deficits in gene and/or encoded protein levels of Complex I-V of the mitochondrial oxidative phosphorylation pathway in the BF. In the frontal cortex (Fr Ctx) these complexes had concomitant alterations in select gene expression but not of the proteins queried from Complex I-V, suggesting that defects at this time point in the BF are more severe and occur prior to cortical dysfunction within the basocortical circuit. We propose dysregulation within mitochondrial oxidative phosphorylation complexes is an early marker of cognitive decline onset and specifically linked to BFCN degeneration that may propagate pathology throughout cortical memory and executive function circuits in DS and AD.

## Introduction

Down syndrome (DS) is caused by the triplication of human chromosome 21 (HSA21) and is the primary genetic cause of intellectual disability (ID). HSA21 triplication is present in approximately 1 in 700 live births and these individuals exhibit multiple systemic functional deficits, including heart conditions, increased incidence of leukemias, epilepsy, premature aging, and neurological deficits (Bittles et al., 2007; So et al., 2007; Lott, 2012; Presson et al., 2013; Mai et al., 2019). While the lifespan of individuals with DS has increased significantly in the past several decades, healthspan lags appreciably behind (Hill et al., 2003; Bittles et al., 2007; Presson et al., 2013; Dick et al., 2016). Most adults with DS start to exhibit Alzheimer’s disease (AD)-like pathology, including senile plaques, neurofibrillary tangles, synaptic dysfunction, and basal forebrain cholinergic neuron (BFCN) degeneration, with the accompanying cognitive decline, typically by the mid-third decade of life (Mann et al., 1984; Coyle et al., 1988; Beacher et al., 2009; Lott and Dierssen, 2010; Costa, 2012; Lott, 2012; Hartley et al., 2015; Annus et al., 2016). BFCN degeneration is an early pathological feature of both DS and AD and coincides with cognitive decline early in disease onset and throughout progression (Yates et al., 1980; Sendera et al., 2000; Mufson et al., 2002, 2008; Iulita et al., 2014). Recent imaging evidence indicates volume reductions in the basal forebrain (BF) predicts entorhinal cortex loss and cortical spread of degeneration in AD and correlates with AD biomarkers (Grothe et al., 2013; Cavedo et al., 2020; Fernández-Cabello et al., 2020; Teipel et al., 2020), suggesting BF dysfunction is one of the earliest pathological changes during the development of AD.

Mitochondrial oxidative phosphorylation is the main energy source within neurons and is critical for normal brain development and function (Mattson et al., 2008; Hall et al., 2012). Deficits within the oxidative phosphorylation pathway have devastating effects on normal neuronal function (Mattson et al., 2008). The oxidative phosphorylation apparatus has five mitochondrial respiratory chain complexes (Complexes I-V), comprised of NADH-ubiquinone oxidoreductase Complex I, succinate dehydrogenase Complex II, cytochrome bc_L_ Complex III, cytochrome C oxidase Complex IV, and the ATP synthase Complex V (Valenti et al., 2014). Individual mitochondrial complex proteins within the oxidative phosphorylation pathway have been shown to be dysregulated in DS cell culture models (Valenti et al., 2016; Briggs et al., 2017). Little information is currently available on the status of individual oxidative phosphorylation markers in the context of DS *in vivo* (Kim et al., 2000, 2001; Bambrick and Fiskum, 2008). Although little doubt exists that oxidative phosphorylation is critical for normal brain function, oxidative phosphorylation pathway deficits in the DS brain demands further exploration. Indeed, recent evidence suggests evaluating oxidative phosphorylation complex proteins in the cortex of the well-established Ts65Dn mouse model of DS and AD is difficult and variable (Lanzillotta et al., 2021), further highlighting the necessity of additional interrogation of the oxidative phosphorylation pathway in the brain of DS and AD relevant model systems.

There are several mouse models of DS available, however, the Ts65Dn mouse is the most prevalent in terms of use and applicability (Rueda et al., 2012; Ruparelia et al., 2012; Ahmed et al., 2017). The Ts65Dn mouse model recapitulates many of the endophenotypes of DS and AD, including hippocampal-dependent learning and memory deficits, BFCN degeneration and septohippocampal circuit dysfunction, notably CA1 pyramidal neuron and choline acetyltransferase (ChAT) activity deficits (Granholm et al., 2000; Belichenko et al., 2004, 2009; Kelley et al., 2014a,b). Degeneration of the BFCN system, including within the septohippocampal and basocortical pathways, are hallmarks of disease progression in DS and AD and are a primary feature of the Ts65Dn mouse model (Holtzman et al., 1996; Granholm et al., 2000; Hunter et al., 2003a; Strupp et al., 2016). BFCN degeneration begins at approximately 6 months of age (MO) in Ts65Dn mice, and loss of BFCNs and deficits in hippocampal cholinergic innervation are uniformly reported by ~10 MO (Holtzman et al., 1996; Cooper et al., 2001; Hunter et al., 2003b; Contestabile et al., 2006; Powers et al., 2016). Many of the mitochondrial deficits reported in human DS, principally in the periphery (Izzo et al., 2018; Bayona-Bafaluy et al., 2021) are also present in this trisomic model including lower ATP production *in vitro* (Valenti et al., 2016) and metabolic changes in peripheral cell types (Cisterna et al., 2020). Further, genes postulated to be involved in mitochondrial dysfunction, including *Dyrk1a* and *Ets2* (Izzo et al., 2018) are triplicated in human DS and reproduced in the Ts65Dn mouse model. These two genes have recently been shown to be upregulated within BFCNs microisolated from the medial septal nucleus (MSN; Alldred et al., 2021).

Although the oxidative phosphorylation pathway is known to be dysregulated in DS (Helguera et al., 2013; Izzo et al., 2018; Bayona-Bafaluy et al., 2021) and changes in oxidative phosphorylation states have significant deleterious effects on normal neuronal function (Mattson et al., 2008), prior studies lack pathway-based evaluations conducted *in vivo* to understand mechanisms driving this critical pathology. Herein, we utilized the Ts65Dn mouse model to examine connectivity based degeneration deficits in two interconnected brain regions comprising the basocortical system, the BF and Fr Ctx, which are critically impacted in DS and AD and are reflective of age-related cognitive decline and loss of executive function. We examined BF and Fr Ctx levels for oxidative phosphorylation changes at the transcript and encoded protein levels within each oxidative phosphorylation complex. We postulate reductions observed within the basocortical circuit indicate BF degeneration drives oxidative phosphorylation dysregulation and paces or precedes cortical dysfunction associated with DS and AD.

## Materials and Methods

### Mice

Animal protocols were approved by the Nathan Kline Institute/NYU Grossman School of Medicine IACUC in accordance with NIH guidelines. Breeder pairs (female Ts65Dn and male C57Bl/6J Eicher x C3H/HeSnJ F1 mice) were purchased from Jackson Laboratories (Bar Harbor, ME, USA) and mated at the Nathan Kline Institute. Mice were given *ad libitum* food and water access (Alldred et al., 2015a,b). Standard cages contained paper bedding and several objects for enrichment (e.g., plastic igloo, t-tube, and cotton square). Mice were maintained on a 12-h light-dark cycle under temperature- and humidity-controlled conditions. Tail clips were taken and pups were genotyped (Duchon et al., 2011) at weaning (P21) and aged to ~6 MO.

### Tissue Preparation

At ~6 MO, mice were sacrificed for brain tissue accession. Mice were given an overdose of ketamine and xylazine and perfused transcardially with ice-cold 0.15 M phosphate buffer (Alldred et al., 2015a,b, 2018, 2019). Brain tissues were accessed from Ts65Dn (Ts; *n* = 10) and age-matched normal disomic (2N; *n* = 10) male mice, with littermates between 2N and Ts mice used when possible (age range: 5.8–6.4 MO, mean age 6.0 MO). The BF was dissected to enrich for cholinergic neurons in the medial septal/ventral diagonal band region (~Bregma 1.35–0.26) as well as the left Fr Ctx dissected using standard coordinates from the mouse brain atlas (Paxinos and Franklin, 2001). Dissections were either flash-frozen or kept on wet ice for homogenization directly following brain accrual. Tissue was homogenized using ice-cold Tris homogenization buffer [THB; 20 mM Tris-Cl (pH 7.4), 1 mM EGTA, 1 mM EDTA and 0.25 M sucrose] with a protease inhibitor cocktail (1:1,000, I3786, Sigma-Aldrich, St. Louis, MO, USA and 1 mM PMSF, ThermoFisher, Waltham, MA, USA) using 1.5 mm zirconium beads on Beadbug homogenizer (Benchmark Scientific, Sayreville, NJ, USA) for 30 s at 4,000 rpm. Post homogenization, samples were kept on ice and cell debris was spun down at 2,500× g for 5 min at 4°C. The supernatant was aliquoted to fresh tubes for isolation of RNA (done immediately following homogenization) or protein assays (each assay done with fresh aliquots of tissue homogenates stored at −80°C). RNase-free precautions were employed, and solutions were made with 18.2 mega Ohm RNase-free water (Nanopure Diamond, Barnstead, Dubuque, IA, USA).

### RNA Purification

RNA from microdissected regions of mouse tissue from BF and Fr Ctx were purified using the miRNeasy Mini kit (Qiagen, Hilden, Germany) according to manufacturers’ specifications. A DNase digestion was performed twice sequentially before the final washes and RNA purification (Alldred et al., 2021). RNA quality control was performed at a 1:5 dilution to preserve RNA for downstream applications (RNA 6000 pico kit, Agilent, Santa Clara, CA, USA).

### RT-qPCR

Equal amounts of RNA was reverse transcribed in a 50 μl reaction volume to generate cDNA from Fr Ctx and BF tissue from Ts and 2N littermates (*n* = 10 per genotype per brain region) using random hexamers as described previously (Alldred et al., 2012, 2015a,b, 2018, 2019; Bordi et al., 2016). RT-qPCR was performed using 1 μl of cDNA and Taqman PCR primers for select genes from oxidative phosphorylation Complexes I-V along with a mitochondrial rRNA gene (Table 1) to assay samples in triplicate on a real-time qPCR cycler (PikoReal, ThermoFisher) as previously described (Alldred et al., 2008, 2012, 2015a,b, 2018, 2019; Jiang et al., 2010). The ddCT method was used to determine relative gene level differences between genotypes (ABI, 2004; Ginsberg et al., 2010; Jiang et al., 2010). Glucuronidase beta (*Gusb*, Mm01197678_m1*)* and 45S pre-ribosomal RNA *(Rn45s*; Table 1) qPCR products were interrogated for use as controls as they did not show significant changes by genotype within MSN BFCN RNA-seq data (Alldred et al., 2021). *Rn45s* was subsequently selected as the control housekeeping gene. Negative controls consisted of the reaction mixture without input RNA. For each gene, the PCR product synthesis was modeled as a function of genotype, using mixed effects models with random mouse effect to account for the correlation between repeated assays on the same mouse (McCulloch et al., 2011; Alldred et al., 2015a,b, 2018, 2019). Significance was judged at the level α=0.05, two-sided.

**Table 1:** List of TaqMan primers for RT-qPCR analysis.

Gene	TaqMan Primer	Description
*Rn45S*	Rn03928990_g1	Housekeeping, 45S pre-ribosomal RNA
*Mt-Nd1*	Mm04225274_s1	Complex I, mitochondrial NADH dehydrogenase 1
*Mt-Nd4l*	Mm04225294_s1	Complex I, mitochondrial NADH 4L dehydrogenase
*Sdha*	Mm01352360_m1	Complex II, succinate dehydrogenase complex flavoprotein, subunit A
*Mt-Cytβ*	Mm04225271_g1	Complex III, mitochondrial cytochrome b
*Mt-Cox2*	Mm03294838_g1	Complex IV, mitochondrial Cytochrome c oxidase subunit II
*Mt-Atp8*	Mm04225236_g1	Complex V, mitochondrial ATP synthase 8
*Mt-Rnr1*	Mm04260177_s1	Mitochondrial 12S rRNA

### Protein Assays

Protein expression analysis was performed using the WES system (Protein Simple, Santa Clara, CA, USA; Nguyen et al., 2011). Briefly, protein samples were diluted in THB buffer 1:100 (w/v), with 1× final concentration of fluorescent molecular weight marker (provided in the kit) and heated to 50°C for 5 min (as per manufacturers’ recommendation), then cooled to 4°C before loading onto the WES system plate with a molecular weight ladder. All blocking reagents, chemiluminescent substrate, separation, and stacking matrices (Protein Simple) were dispensed to designated wells. Primary antibodies against Complex I-V (Total OXPHOS panel, 1:20 dilution, ab110413, AbCam, Cambridge, United Kingdom) containing five mouse mAbs, Complex I (NADH:ubiquinone oxidoreductase subunit B8; NDUFB8), Complex II (succinate dehydrogenase beta; SDHB), Complex III (Cytochrome b-c1 complex subunit 2;UQCR2), Complex IV (Mitochondrially encoded cytochrome C oxidase I; MTCO1) and Complex V (ATP synthase lipid-binding protein; ATP5A), along with a control antibody against β-Tubulin III (β-TubIII; R&D Systems, Minneapolis, MN, USA, MAB1195 1:50) and HRP conjugated secondary antibody (rabbit anti-mouse; DM-002; Protein Simple) were dispensed to designated wells. Plates were spun for 5 min at 1,000× *g* and loaded onto the WES unit, where separation electrophoresis and immunodetection steps are fully automated within the capillary system. Instrument default settings were used with the exception of protein loading run time that was increased from 25 to 35 min. The digital image was analyzed with Compass software (Protein Simple), utilizing dropped lines for peak analysis area calculation. Detected proteins were compared to control protein (β-TubIII) and reported as the normalized percentage of control. Each protein was performed in triplicate on separate plate runs. Statistical analysis was conducted on each protein normalized to β-TubIII and modeled as a function of the mouse study group (Ts and 2N, *n* = 10 per genotype per brain region), using mixed effects models with random mouse effect to account for the correlation between repeated assays on the same mouse (McCulloch et al., 2011; Alldred et al., 2015a,b, 2018, 2019). Significance was judged at the level *α* = 0.05, two-sided.

### Deproteinization and ATP Assay

Biochemical analysis of ATP levels were conducted using Fr Ctx tissue. Deproteinization of mouse Fr Ctx homogenates were performed utilizing the Deproteinizing Sample preparation kit (ab204708, AbCam) according to manufacturer’s specifications with the following alterations, starting sample volume was reduced to 50 μl from 100 μl with 7.5 μl of TCA, and neutralization was performed with 5 μl of neutralization solution. Immediately following deproteinization, an ATP assay (ATP assay kit, ab83355, AbCam) was performed in duplicate for each sample (*n* = 6 per genotype) utilizing a 1:4 dilution of the deproteinized sample according to the manufacturer’s specifications for the fluorometric assay. The fluorometric samples were read in duplicate on a plate reader (SpectraMax, Molecular Devices, San Jose, CA, USA). ATP concentration was calculated according to the manufacturer’s specifications. Statistical analysis was performed using a non-parametric method (Wilcoxon Test) due to the small sample size and some losses of the sample (Wilcoxon, 1946; Pratt, 1959). Significance was judged at the level *α* = 0.05, two-sided.

## Results

**Figure 1:**
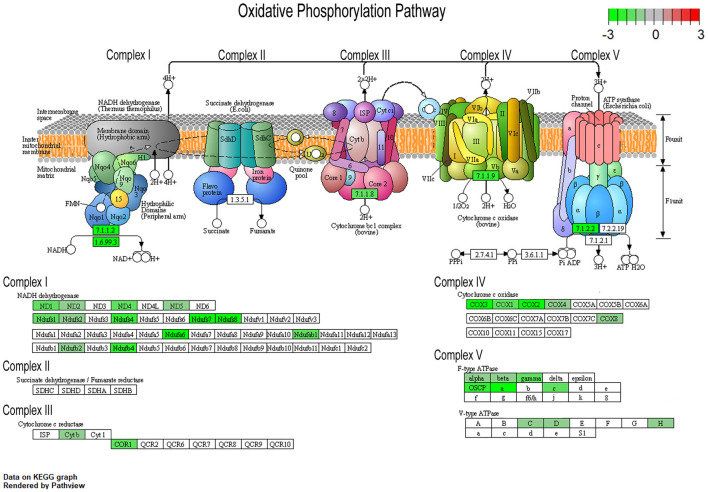
Pronounced downregulation of members of the oxidative phosphorylation pathway in trisomic basal forebrain cholinergic neurons (BFCNs). The Kyoto Encyclopedia of Genes and Genomes (KEGG) derived oxidative phosphorylation pathway is rendered by Pathview, with significantly dysregulated genes highlighted for each complex from the medial septal nucleus (MSN) BFCNs as assayed by single population RNA-seq (Alldred et al., 2021). Downregulation is shown as color coded log fold change difference (LFC) with green depicting downregulation and red depicting upregulation. Note no genes within the oxidative phosphorylation pathway were upregulated.

To understand changes in oxidative phosphorylation within the basocortical circuit, we examined RNA and protein levels from each of the five complexes in the oxidative phosphorylation pathway from two synaptically connected brain regions critical for attention, memory, and executive function that show a significant decline in DS and AD. This examination was based upon gene expression profile changes in the oxidative phosphorylation pathway from MSN BFCNs by single population RNA-seq in ~6 MO Ts65Dn mice compared to 2N littermates (Alldred et al., 2021). Bioinformatic inquiry by Kyoto Encyclopedia of Genes and Genomes (KEGG) analysis of the oxidative phosphorylation pathway revealed downregulation of multiple subunits of Complex I, III, IV, and V, but no significant changes in Complex II subunits within MSN BFCNs (Figure 1).

**Figure 2:**
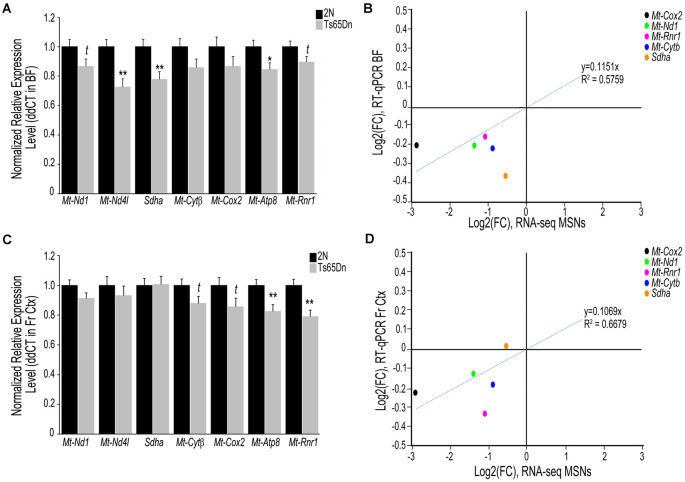
Interrogation of oxidative phosphorylation Complex I-V subunit gene expression within the trisomic basocortical circuit. RT-qPCR was performed to determine gene expression levels using regional dissections of the basal forebrain (BF; **A,B**) and Fr Ctx **(C,D)** from Ts65Dn and 2N littermates at ~6 MO for six genes. **(A)** Bar graph represents ddCT of each gene normalized to 2N levels in the BF. Significant downregulation was found for *Mt-Nd4l, Sdha*, and *Mt-Atp8* and trend-level downregulation was found for *Mt-Nd1*, *Mt-Cytβ*, and *Mt-Rnr1*. **(B)** Correlation plot association between MSN BFCN RNA-seq LFC (x-axis) and BF RT-qPCR LFC (y-axis). **(C)** Bar graph represents ddCT of each gene normalized to 2N levels in the Fr Ctx. Significant downregulation was found for *Mt-Atp8* and *Mt-Rnr1* and trend-level downregulation was found for *Mt-Cytβ* and *Mt-Cox2*. **(D)** Correlation plot association between MSN BFCN RNA-seq LFC (x-axis) and Fr Ctx RT-qPCR LFC (y-axis). While most genes were not significantly downregulated by RT-qPCR, they trended in the same direction (downregulation). *Sdha* was the only gene that did not correlate with RNA-seq results. Black bars represent relative 2N expression and gray bars indicate Ts65Dn expression normalized to 2N for each gene (standard error of mean (SEM) is indicated by error bars). Key: *p < 0.05, **p < 0.01; *t*, trend.

To examine if transcriptomic changes seen in the Ts65Dn MSN BFCNs are selective to the MSN enriched BF or are seen throughout the basocortical circuit, we examined both BF and Fr Ctx at the RNA and protein level for expression changes at the start of BFCN degeneration. We examined a total of six subunits from Complexes I-V within the oxidative phosphorylation pathway for gene expression, utilizing transcripts that were significantly downregulated in MSN BFCNs *via* single population RNA-seq along with candidates that were not differentially regulated, to examine the pathway in a comprehensive manner. We also assessed the *Mt-Rnr1* gene, which encodes the 12S rRNA as a marker of total mitochondrial RNA. In the BF, downregulation was observed for members of Complex I, *Mt-Nd1* (trend level *p* = 0.072) and *Mt-Nd4l* (*p* < 0.00228), Complex II, *Sdha* (*p* < 0.00846), Complex III, *Mt-Cytβ* (not significant), Complex IV, *Mt-Cox2* (not significant), and Complex V, *Mt-Atp8* (*p* < 0.0268; Figure 2A). Total mitochondrial rRNA (*Mt-Rnr1*) also showed a downregulated trend (*p* = 0.064; Figure 2A). When correlating the single population MSN BFCN RNA-seq to regional BF RT-qPCR log fold changes (LFC), a moderately high correlation was observed (*R*^2^ = 0.5759; Figure 2B). Interestingly, while not significant, downregulation observed *via* RT-qPCR for *Mt-Nd1* and *Mt-Cox2* correlated with the significant downregulation seen in trisomic MSN BFCNs by RNA-seq. The regional BF gene expression of *Mt-Nd1* was only trend level downregulated *via* RT-qPCR, suggesting these changes are neuron-specific and the observed downregulation within MSN BFCNs is diluted when examined in tissue with admixed neuronal and non-neuronal cell types. The same subset of genes was interrogated in the Fr Ctx of Ts65Dn mice compared to 2N littermates by RT-qPCR. Of the six genes examined from the five complexes, no changes were found in either Complex I or II genes (*Mt-Nd1*, *Mt-Nd4l*, and *Sdha*), trend level decreases in Complex III and IV genes (*Mt-Cytβ* *p* = 0.067 and *Mt-Cox*2 *p* = 0.079), and significant downregulation of Complex V member *Mt-Atp8* (*p* < 0.0059; Figure 2C). Total mitochondrial rRNA was significantly downregulated in the trisomic Fr Ctx (*p* < 0.0012; Figure 2C). Similar to regional BF RT-qPCR, a moderately high correlation between MSN BFCN RNA-seq and Fr Ctx RT-qPCR LFC was found (*R*^2^ = 0.6679; Figure 2D).

**Figure 3:**
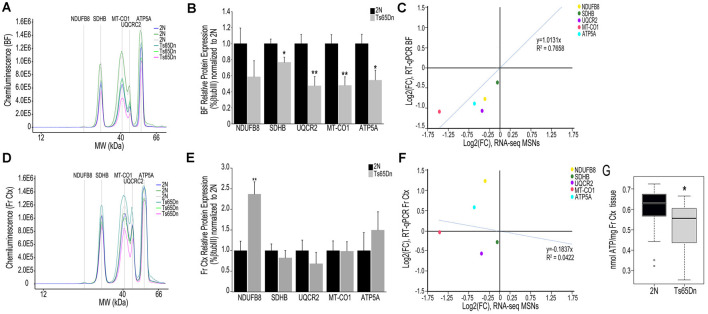
Interrogation of oxidative phosphorylation Complex I-V subunit protein expression within the trisomic basocortical circuit. **(A)** Representative digital signatures of each assayed protein raw expression levels (y-axis) and molecular weight (x-axis) from Ts65Dn and 2N BF tissue homogenates. **(B)** Bar graph represents relative protein levels (normalized to beta-tubulin (βTubIII) as a percentage of 2N expression in BF tissue homogenates. Black bars represent 2N and gray bars indicate Ts65Dn (SEM indicated by error bars). **(C)** Correlation plot association between MSN BFCN RNA-seq LFC (x-axis) and BF protein level LFC (y-axis) indicating a high correlation. **(D)** Representative digital signatures of each assayed protein raw expression levels (y-axis) and molecular weight (x-axis) from Ts65Dn and 2N Fr Ctx tissue homogenates. **(E)** Bar graph represents relative protein levels (normalized to βTubIII) as a percentage of 2N expression in Fr Ctx tissue homogenates (SEM indicated by error bars). **(F)** Correlation plot association between MSN BFCN RNA-seq LFC (x-axis) and Fr Ctx protein level LFC (y-axis) indicating no significant correlation. **(G)** Box and whisker plots highlight downregulation of ATP levels in trisomic Fr Ctx. Key: *p < 0.05, **p < 0.01.

We employed the Total OXPHOS panel (Abcam) to assess protein expression of select subunits from Complexes I-V in the BF and Fr Ctx within Ts65Dn mice and 2N littermates. When examining the digital traces in BF homogenates (Figure 3A), we saw a relatively high expression of Complex II-V proteins, but relatively low expression of Complex I protein (NDUFB8), indicating this may not be a major subunit expressed in basocortical brain tissue. Within the BF, Complex I, NDUFB8 expression was not significantly downregulated. However, downregulation of proteins from Complex II (SDHB, *p* < 0.016), III (UQCRC2, *p* < 0.0055), IV (MT-CO1, *p* < 0.0034), and V (ATP5A, *p* < 0.019) were found (Figure 3B), which highly correlated with MSN BFCN RNA-seq data (*R*^2^ = 0.7658; Figure 3C). When examining the same Complex I-V proteins in Fr Ctx tissue, variability in relative expression levels was observed between animals (Figure 3D) and no significant downregulation of protein expression was detected. The only significant change in protein expression in trisomic Fr Ctx tissue homogenates was upregulation of Complex I (NDUFB8, *p* < 0.0012; Figure 3E). In contrast to RT-qPCR findings, protein levels in Fr Ctx did not correlate with MSN BFCN RNA-seq data (*R*^2^ = 0.0422, Figure 3F).

We indirectly examined the functionality of the oxidative phosphorylation complex by biochemical analysis of ATP levels, which are the output of the ATP synthase Complex V. Due to the small size of mouse BF and the quantity of tissue needed for the ATP assay, we were only able to perform this measurement in Fr Ctx tissue. We found a significant decrease in ATP levels in trisomic Fr Ctx (*p* < 0.0307; Figure 3G), which matched Fr Ctx RT-qPCR findings for Complex V.

## Discussion

We employed the Ts65Dn mouse model of DS and AD to examine oxidative phosphorylation pathway changes within the basocortical circuit. The BF provides the main cholinergic inputs for the hippocampus and cerebral cortex (Mesulam et al., 1983). Cholinergic fiber input into the cortex is involved in both attentional behavior and cognition and the activity of these cholinergic neurons is decreased during normal human aging (Mufson et al., 2003). This deficit is exacerbated during AD and DS progression (Coyle et al., 1986, 1988; Mufson et al., 2003). We examined expression level differences of individual mitochondrial oxidative phosphorylation subunits at the transcript and encoded protein levels ~6 MO, a timepoint where BFCN degeneration is initiated in Ts65Dn mice. Bioinformatic inquiry of our recent single population RNA-seq analysis of MSN BFCNs in Ts65Dn and 2N littermates indicates that oxidative phosphorylation and mitochondrial dysfunction are two of the top canonical pathways by Ingenuity Pathway Analysis (IPA) downregulated in this vulnerable cell type directly relevant to human DS and AD pathophysiology (Alldred et al., 2021). Understanding the age of degeneration onset and the pathways involved at this inception point would help pinpoint novel targets for therapeutic development for slowing or stopping the degeneration associated with cognitive decline and loss of executive function. Indeed, despite hundreds of trials for AD and DS treatment, no new therapeutics have proven effective for AD or DS, with researchers suggesting that the delayed onset of treatment is a driving factor of the failure of these clinical trials (Gauthier et al., 2016; Yiannopoulou et al., 2019).

Oxidative stress and mitochondrial dysfunction are thought to play a critical role in DS and AD pathology (Mattson et al., 2008; Lott, 2012; Helguera et al., 2013; Izzo et al., 2018). Interestingly, a recent review postulates DS is an oxidative phosphorylation disorder (Bayona-Bafaluy et al., 2021). To date, the majority of studies evaluating oxidative phosphorylation and mitochondrial dysfunction in DS have used *in vitro* model systems. Although helpful to ascertain mechanistic interactions, these *in vitro* studies benefit from parallel *in vivo* assessments using the animal model and postmortem human brain tissue in the context of DS and AD for greater applicability and disease relevance.

Based on the present results, we postulate downregulation of select members of the oxidative phosphorylation pathway in the BF precedes degenerative changes within Fr Ctx, in a connectivity based degeneration course of action. Supporting evidence comes from the observation that downregulation of oxidative phosphorylation pathway complex genes is less pervasive in Fr Ctx along with a generalized lack of encoded protein changes at ~6 MO, whereas the BF has profound transcript and protein level changes (Figures 2A,C, 3B,E). These results correlate strongly with our previous RNA-seq analysis (Figures 2B, 3B) within MSN BFCNs, indicating mitochondrial oxidative phosphorylation complexes are highly vulnerable in DS. Complementary observations were found in the Fr Ctx by an independent group using the Ts65Dn model at different age timepoints including no changes in Complex II (SDHB) or Complex V (ATP5A) subunits and with deficits in Complex III (UQCRC2) and Complex IV (MT-COX2) not seen until 18 MO (Lanzillotta et al., 2021). Our ~6 MO data is commensurate with their 9 MO data showing a significant increase in Complex I (NDUFB8) levels, which is reversed at 18 MO, possibly indicating data this independent research group postulates is due to the shift from juvenile age to adulthood and to aged animals, whereby Ts65Dn mice react differently than their 2N counterparts (Lanzillotta et al., 2021). These researchers also conclude the oxidative phosphorylation machinery is highly downregulated in their DS cohort (Lanzillotta et al., 2021). BF was not evaluated, so comparisons are only available in the Fr Ctx. Although a compensatory mechanism prior to overt pathology onset is possible and would explain upregulation of NDUFB8 protein levels in Fr Ctx, differential regulation of this subunit does not reflect overall deficits in the oxidative phosphorylation pathway seen in our ~6 MO trisomic cohort during initiation of BFCN degeneration. We also demonstrate downregulation of cholinergic and glutamatergic protein levels in the BF (Supplementary Figure 1), indicating synaptic deficits also exist in ~6 MO trisomic mice, correlating with single population RNA-seq findings (Alldred et al., 2021). Synaptic-related marker downregulation in the BF which project to the Fr Ctx, along with the high number of significantly downregulated genes and proteins evidenced in BF, and the reduction of corresponding deficits in Fr Ctx are supportive of our overarching hypothesis that BF degeneration drives oxidative phosphorylation dysregulation and precedes cortical dysfunction in DS as well as across the AD spectrum (Mufson et al., 2016, 2019). Deficits in the activity of the oxidative phosphorylation complex have been shown in astrocyte cultures derived from the Ts1Cje DS mouse model, including lower ATP levels and decreased mitochondrial membrane potential (Shukkur et al., 2006). This study also showed *in vivo* brain ATP levels are reduced in Ts1Cje mice at 3 MO (Shukkur et al., 2006). Neural progenitor cells obtained from the Ts65Dn hippocampus revealed reduced ATP levels and loss of mtDNA levels, indicating a neuronal deficit in energy production (Vacca et al., 2016; Valenti et al., 2018). We corroborate these findings within the Ts65Dn model *in vivo*, with reduction in ATP levels and overall mitochondrial RNA (*via* *Mt-Rnr1*) in Fr Ctx (Figures 2C, 3G). We postulate significant decreases in trisomic mitochondrial RNA, as determined by *Mt-Rnr1* may drive gene expression changes seen in Fr Ctx tissue. Interestingly, these deficits may be the result of the use of regional tissue with admixed cell types for the protein assays, with the expectation that neuron-specific protein level changes may be obscured. Conversely, BF oxidative phosphorylation changes are likely to involve multiple neuronal subtypes within the BF and are able to be discriminated even in admixed tissue.

Mitochondrial dysfunction beyond the oxidative phosphorylation pathway has been linked to DS pathology including deficits in mitochondrial biogenesis, turnover, and mitophagy (Helguera et al., 2013; Izzo et al., 2018; Bordi et al., 2019; Mollo et al., 2019, 2020). The balance between biogenesis and mitophagy is perturbed using *in vitro* models of DS, with hyperactivation of proteins from the rapamycin (mTOR) pathway responsible for mitophagy and impaired activity of PGC-1α pathway responsible for biogenesis (Valenti et al., 2016; Bordi et al., 2019; Mollo et al., 2019, 2020). These studies link dysregulation of mitochondrial function to dysregulation of autophagy pathways, which have also been shown to be dysregulated in MSN BFCN neurons by single population RNA-seq (Alldred et al., 2021). We postulate BFCN degeneration in DS and AD may be directly related to the failure of mitochondrial turnover and mitophagy. From a translational perspective, choline, an essential nutrient required for the production of the cholinergic neurotransmitter acetylcholine, the phosphatidylethanolamine N-methyltransferase (PEMT) pathway for generation of key substrates of neuronal membranes, and the primary methyl donor in the brain, requires properly functioning mitochondria and the oxidative phosphorylation pathway (Mailloux et al., 2016). Our collaborative group demonstrated metabolites of the PEMT pathway are significantly downregulated in the brains of Ts65Dn mice including within the BF and Fr Ctx (Yan et al., 2014), which also implicate deficits in mitochondria and the oxidative phosphorylation pathway. Further study on overall mitochondrial turnover, morphology, and mitophagy within *in vivo* DS and AD models are warranted.

RT-qPCR analysis and protein chemistry in trisomic BF and Fr Ctx reveals downregulation within oxidative phosphorylation genes and proteins that are specific to the brain regions analyzed. We recognize there are limitations and caveats to the studies we conducted using this approach. For example, each of these complexes in the oxidative phosphorylation pathway consists of numerous subunits, and the current interrogation was limited to one to two genes or proteins per complex. It is possible that other Complex I-V subunits will not be differentially regulated, although the larger number of changes identified by IPA and KEGG analysis from the MSN BFCN RNA-seq study of ~6 MO Ts65Dn mice and 2N littermates analysis indicates BFCNs are significantly vulnerable and display an abundance of deficits in oxidative phosphorylation and mitochondrial function transcripts (Alldred et al., 2021). Another noted limitation is that when examining the correlation analysis between BF regional RT-qPCR and single population MSN BFCN RNA-seq, we were unable to compare *Mt-Nd4l* or *Mt-Atp8*, as they were not detected by RNA-seq (Alldred et al., 2021), likely due to the small size of the full-length mitochondrial RNA and low RNA input (McCormick et al., 2011; Stark et al., 2019). It is important to note this study was performed in male mice, and sex differences may exist in BFCN degenerative programs, as morphological differences between sexes in BFCNs have been demonstrated in trisomic mice (Kelley et al., 2014b). A cohort of female trisomic mice is currently being accrued for RNA-seq, RT-qPCR, and protein-based analyses, enabling sex differences to be evaluated in future studies. Importantly, Lanzillotta et al. (2021) demonstrate expression level changes in the oxidative phosphorylation pathway in Fr Ctx within trisomic mice during aging. Although beyond the scope of the present study, an aging time-course assessment of the Ts65Dn BF is warranted in future studies. Moreover, future assessments are planned to evaluate vulnerable cell types, brain regions, age, and sex in trisomic models in relation to parallel observations found in human postmortem DS and AD studies in the same cell types and regions.

In conclusion, select dysregulation of oxidative phosphorylation pathway members is found at the RNA and encoded protein levels within the vulnerable basocortical circuit in an established model of DS and AD at a timepoint where BFCN degeneration is occurring. Defects appear to initiate in the BF and travel in the synaptic pathway that connects this vulnerable region to a cortical terminal field associated with memory and executive function. These data suggest deficits in oxidative phosphorylation in the DS and possibly AD brain may be circuit driven, and more specific to vulnerable brain regions than previously appreciated, especially in the context of neuropathological disorders and age–related cognitive decline.

## Data Availability Statement

The raw data supporting the conclusions of this article will be made available by the authors, without undue reservation.

## Ethics Statement

The animal study was reviewed and approved by IACUC Nathan Kline Institute.

## Author Contributions

MJA GES, and SDG designed the experiments. MJA performed experiments. SHL, MJA, and SDG performed analysis of data. MJA and SDG wrote the manuscript. All authors contributed to the article and approved the submitted version.

## Conflict of Interest

The authors declare that the research was conducted in the absence of any commercial or financial relationships that could be construed as a potential conflict of interest.

## Publisher’s Note

All claims expressed in this article are solely those of the authors and do not necessarily represent those of their affiliated organizations, or those of the publisher, the editors and the reviewers. Any product that may be evaluated in this article, or claim that may be made by its manufacturer, is not guaranteed or endorsed by the publisher.
