# Supplementary figures and images for: Oxidative Phosphorylation Is Dysregulated Within the Basocortical Circuit in a 6-month old Mouse Model of Down Syndrome and Alzheimer’s Disease
Source: Front Aging Neurosci. 2021 Aug 19;13:707950. doi: 10.3389/fnagi.2021.707950 (PMC8417045; doi:10.3389/fnagi.2021.707950)

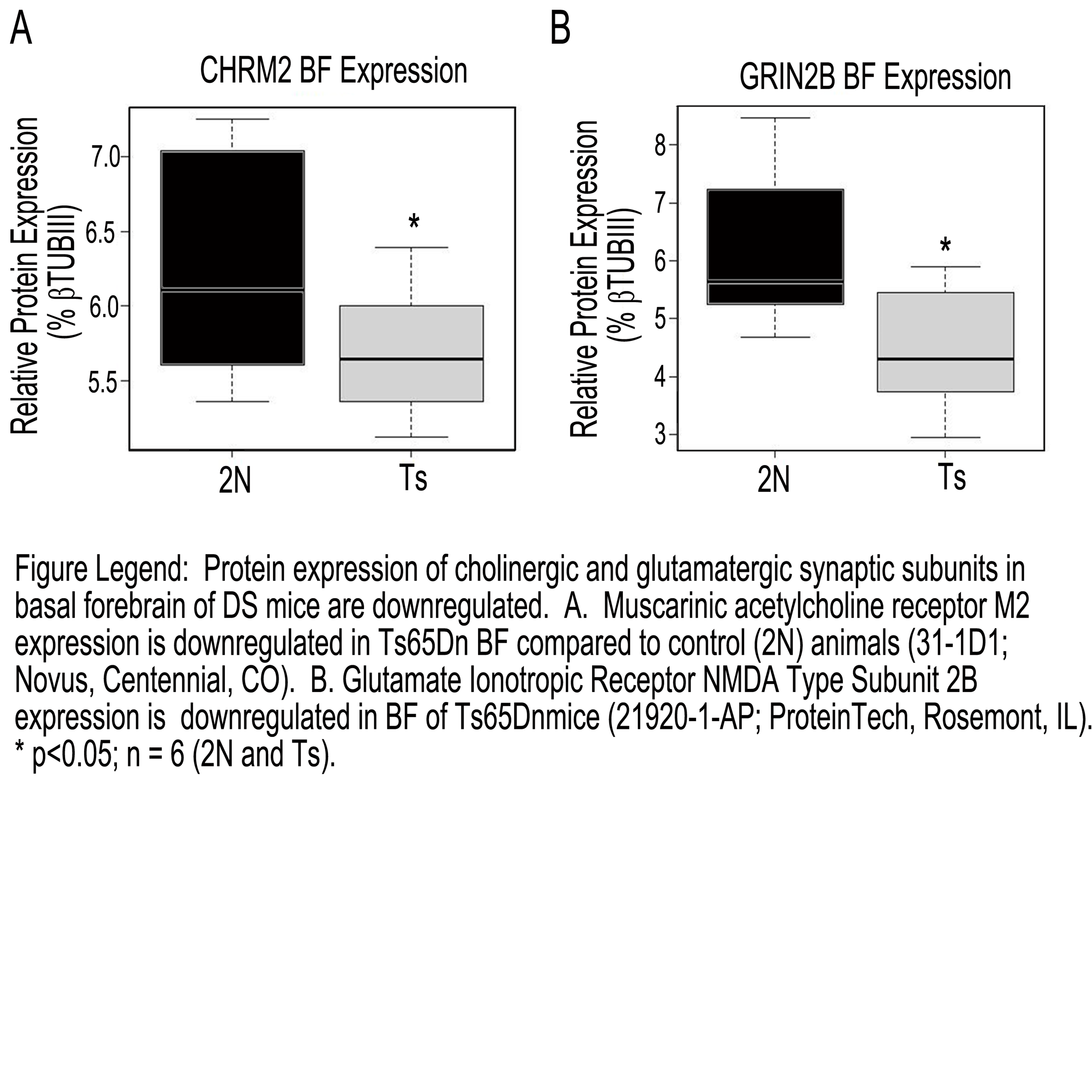

Supplement: Supplementary file 1 [file Image_1.TIF]
